# Obsessive Compulsive Disorder: Frequency and Gender Estimates

**DOI:** 10.12669/pjms.36.5.1870

**Published:** 2020

**Authors:** Shaista Jabeen, Rukhsana Kausar

**Affiliations:** 1 Dr. Shaista Jabeen, PhD., Applied Psychology (Clinical Psychology). Assistant Professor, Department of Psychology, Forman Christian College University, Lahore, Pakistan; 2 Prof. Dr. Rukhsana Kausar, PhD & Post Doc. (UK). Director, Vice Chancellor, Govt. College Women University, Sialkot, Pakistan.

**Keywords:** OCD, Frequency, Gender difference

## Abstract

**Objective:**

Frequency data on mental disorders is a crucial requirement for primary, secondary and tertiary prevention. However, such local data is sparse. The study aimed to estimate frequency and gender differences of Obsessive Compulsive Disorder (OCD) in clinical population.

**Method:**

Retrospective data of adult patients (18 years and above) reported in Out Patient Departments (OPD) of four teaching hospitals and a private clinic was collected. Successive three years record was consulted using OPD registers. Gender difference in frequency of OCD was estimated.

**Results:**

Altogether 90119 patients were registered in five psychiatric settings in major cities of Punjab during three year period. As information was missing for over one third of the registered patients, hence analysis was carried out on valid cases only i.e. 59220 (65.8% of the total number of patients registered). It was revealed that the estimated frequency of OCD is 4.1%. There was no significant gender difference revealed in frequency of OCD (P>.05)

**Conclusion:**

Frequency of OCD implicate the need for early detection and intervention of the disorder. Further, it elucidates the importance of community based research on other mental disorders. The need for accurate record keeping which is a core element for any research related with medical or psychological issue is also highlighted.

## INTRODUCTION

Obsessive Compulsive Disorder (OCD) is quite common psychiatric disorder which affects quality of life of the sufferer. It is linked with high levels of social and occupational impairment,^1^ hence highlighting the need to explore this clinically significant area. It has been reported to be the fourth most common psychiatric disorder in the United States.^2^ Prevalence of OCD in clinical settings is three times lower than the estimates based on community epidemiological studies, hence rendering OCD as under representative in clinical settings.^3^ Moreover, Patients presenting with OCD are most likely to be treated in outpatient settings and are often not admitted^4^ hence the number of admissions of patients with OCD turns out quite low in the indigenous literature.^5^

Prevalence estimates of OCD vary across the world. Twelve month prevalence of OCD has been reported to be 1.2% in the US and between 1.1 - 1.8% globly.^1^ Studies carried out in different regions of Asia i.e. Iran and Singapore revealed a life time prevalence of 1.8% and 3% respectively.^6^,^7^ South Asian studies indicate prevalence rates of OCD ranging from 0.28% in India, 3% in Pakistan.^8^,^9^ In Turkey OCD is prevalent in 3% in general public to 4.2% in university students.^10^,^11^

With regard to male female ratio of OCD, women presented with slightly higher rates compared to men in University students in Turkey (i.e. 3.3% vs. 2.5%).^11^ Ratio of lifetime and 12-month prevalence for male: female of any anxiety disorder was 1:1.7 and 1:1.79, respectively. Women reported to have higher rates of lifetime diagnosis for most of the anxiety disorders including OCD. Women with anxiety disorders caused more illness burden compared to men.^12^

Literature on prevalence of OCD in the general population in Pakistan and estimation of gender based data in Punjab, Pakistan is sparse. Located in South Asia, Pakistan is the second largest Muslim country. It has five provinces and Azad Kashmir region. Punjab is Pakistan’s highest populous province while it is the second largest province in terms of area. However the data available to indicate the prevalence of OCD in Punjab is meager. OCD is closely linked with diminished quality across various domains of life when compared to normative data. Temporal studies of OCD indicate that the impacted quality of life of OCD sufferers improves after pharmacotherapy or cognitive behaviour therapy or both. The serious impact of OCD on persons’ quality of life, their emotional, social and mental wellbeing and burden of disease summon the need to conduct such study to identify the magnitude of the problem.

Further, community prevalence studies on the subject are important and much needed. Since no such local studies are available, the frequency noted in hospitalized patients gives some idea about the burden of the disorder. The current study is designed to explore the frequency and gender differences of OCD in different hospitals of Punjab.

## METHODS

The research was conducted in three phases: During the first phase, the researcher approached various teaching hospitals (11 Government Hospitals and five private clinics) in Punjab province. Most of the private clinics declined access to the data. Overall, the researcher was successful in attaining permission to collect data from four Government hospitals and one private clinic. Confidentiality of individual patients’ information was ensured.

Subsequent to seeking approval from the concerned heads of the psychiatry departments to collect data, the researcher was provided access to the record rooms to consult the last three years record. All efforts were made to understand the recorded information including requesting the medical officer on duty for helping to read the diagnoses correctly. Data of the adult patients including men and women (above age 18 years) attending outdoor services with a diagnosis of OCD were included in the sample.

Third phase comprised of the process of data scanning and data sorting for analysis. The same procedure was followed for all the hospitals included in the study. Some of the data with missing information (e.g. age, diagnosis and gender) had to be discarded. In some cases, it was not clear that the diagnosis of OCD was provisional or final. All data with sketchy information or missing information were excluded as it did not help in their count due to lack of information pertaining to the inclusion criteria.

The information about new or old patients was also missing in many cases and the researcher had to compromise to include all the cases. In some cases, the data was not recorded for some practical administrative reasons reflecting the low number of patients who had availed of the service.

**Fig.1:**
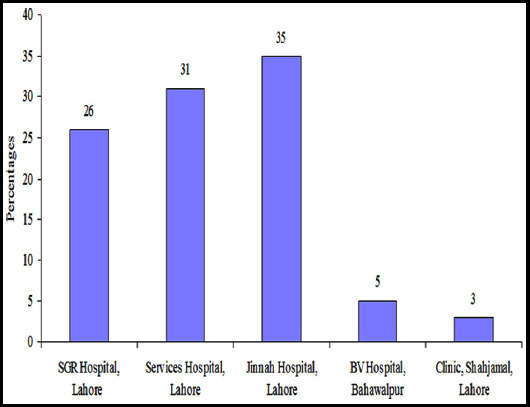
Frequency of OCD cases per hospital (Total N= 2441).

## RESULTS

Frequency of OCD were estimated by comparing the number of patients presented in each hospital with the total number of patients in three successive years. Of the total reported cases (90119), only 59220 (65.7%) qualified to be valid for further analysis as information pertaining to diagnosis, age and gender was missing in rest of the cases i.e. 30861 (34.3% of the total registered cases). Percentages of valid cases in each individual clinical setting is shown in Table-I.

**Table-I:** Hospital wise Distribution of Patients presented in different Hospitals during 3 years.

Hospitals	Men	Women	Total	Valid Cases (%)		

				Male	Female	Total
Sir Ganga Ram Lahore	6317	6059	12376	5807	5613	11420 (92)
Services Hospital Lahore	12250	9243	21493	4317	3512	7829 (36)
Jinnah Hospital Lahore	11534	11138	22672	9254	9375	18629 (82)
BV* Hospital Bahawalpur	14346	16916	31262	9237	9967	19204 (61)
Shah Jamal Clinic Lahore	1372	944	2316	1256	882	2138 (92.3)

Total	45819	44300	90119	29871	29349	59220 (65.7)

* BV= Bahawalpur Victoria.

The remaining analysis is based on valid cases only i.e. 59220 cases only (i.e. 65.7% of the total cases). Of which 29870 (50.4%) were men and 29349 (49.6%) were women.

Hospital wise distribution of the total number of reported cases and the cases diagnosed with OCD per hospital in the study is shown in Table-II. Amongst teaching hospitals, in spite of having the highest number of valid cases, BV Hospital, Bahawalpur had the lowest percentage of OCD cases. On the other hand Services Hospital, Lahore has the lowest number of valid cases but had the highest number of patients diagnosed with OCD. On average 4% of the valid cases were diagnosed with OCD.

**Table-II:** Patients with OCD reported in different Hospitals in Punjab during 3 Years.

Hospitals/Clinic	Valid registered patients	f		Hospital wise OCD cases (%)

		Male	Females	
SGR Hospital, Lahore	11421	309	321	630 (5.5)
Services Hospital, Lahore	7828	346	409	755 (9.6)
Jinnah Hospital, Lahore	18629	492	367	859 (4.6)
Bahawal Victoria (BV) Hospital, Lahore	19204	49	70	119 (0.6)
Clinic, Shahjamal, Lahore	2138	50	28	78 (3.6)

Total	59220	1246	1195	2441 (4.1)

**Table-III:** Gender differences in Frequency of OCD in different Hospitals in Punjab (N= 2441).

Number of patients with OCD (%)				Ratio	x^2^	P

Male		Female				

*ƒ*	*%*	*ƒ*	*%*			
1246	4.17	1195	4.07	1:0.95	1.06	0.30

Of the five hospitals studied, Jinnah Hospital Lahore outnumbered the cases diagnosed with OCD in teaching hospitals making over 35% of the total number of the OCD cases reported while BV hospital represented the smallest number of OCD patients amongst teaching hospitals in the study.

Chi Square test indicates no significant gender difference in the frequency of OCD in clinical settings studied.

## DISCUSSION

Patients’ hospital records of three years were explored to estimate frequency of OCD in clinical settings. Hospital records have been used in the studies outside Pakistan to estimate prevalence and incidence rates of OCD.^3^ Data of large prevalence number of cases had to be discarded due to lack of information required for the study (34.3%). Bahawalpur Victoria (BV) Hospital, Bahawalpur came out having the highest turn out of patients. This is one of the largest hospitals in the Punjab province providing psychiatric facilities to Bahawalpur district and its vicinities. However despite catering the maximum number of psychiatric cases, data indicates that BV Hospital, Bahawalpur reported the lowest number of OCD patients. Overall rates of OCD in all of the five psychiatric settings range from 3.2% (in the private clinic) to 35.2 % (in Jinnah Hospital, Lahore). Lahore based hospitals altogether made up 95% frequency of the total OCD cases in Punjab. Obsessive Compulsive Disorder is extremely under reported in BV Hospital when compared to the rest of the Government hospitals of Punjab in the sample. The considerably low frequency of OCD in Psychiatry Department of BV hospital, Bahawalpur which caters mental health needs of patients mostly from South Punjab may be attributed to educational, economical, cultural and psychological factors. Lack of awareness about psychological problems and their management, secretive nature of the problem as well as feelings of shame and embarrassment associated with debilitating disorder of OCD can also be contributing factors to low frequency. Moreover, in case of female patients, the availability and motivation of a significant family member to bring them for psychiatric consultation is another cultural factor which may have resulted in low attendance in the facilities reflecting low frequency in that particular health facility. A register based study in a Healthcare Department in Northern Finland attributed low rates of referrals for OCD due to regional and social class differences and lack of awareness.^13^ Low social class and low education have been associated with overall adverse mental health and low visits to mental health facilities.^14^,^15^

In the current study high frequency of OCD in Lahore based hospitals may be due to higher literacy rates and better level of awareness of mental health issues in the general public in the vicinity of Lahore. However, the results impacted due to use of different diagnostic approaches across settings cannot be ruled out. For instance, point prevalence of OCD was reported to be 3.3% among college students in India^16^ in contrast to the findings of a study of senior high school adolescents reporting prevalence of OCD as 1.39% in Greece.^17^ It is pertinent to note that different methodologies used in population as well as clinical studies to estimate the prevalence rates may have rendered impact on the reported OCD rates.

Due to lack of clinical based literature on frequency of OCD, reference has been made to community based studies to observe the similarities and diversities in this research. Although population based data in Pakistan are scarce, rates of 3% of OCD reported in general population^9^ and the current statistics based on hospital record indicate that the number of people suffering from OCD who require psychiatric input is quite large. Findings indicate that both men and women can equally suffer with this ailment. Findings of the present study coincide with another South Asian study where no gender differences were found in the prevalence of OCD in India.^16^ However, there are both previous and recent studies reporting varied reports of preponderance of men or women presenting with OCD. This variation is supported by predominance of female population presenting with OCD in Iran^6^ as well as in Greece.^18^ On the other hand, data from Egypt predominantly a Muslim country reveals that more than two third of the OCD sample were men pointing to a complex interaction of cultural and religious aspect of the disorder and supporting the variation in gender representation.^19^ Findings highlight the need for further research investigating different aspects of OCD. The fact that many people though in need of help, may not be utilizing the available treatment facilities due to cultural, economical, social and logistic factors cannot be ignored.

### Limitation of the study

It was not possible to identify old or new cases from the hospital registers; hence the findings should be interpreted keeping this fact in view. It is important to note that different diagnostic criterion was used to diagnose patients with OCD across different settings so a single criterion cannot be identified as a benchmark. Further, the study is based on clinical population hence results cannot be generalized to general population.

### Future Implications

The study highlights the need to do research on general population to come up with the prevalence rates of OCD and other psychiatric disorders in the community. The study on a bigger scale using large sample will help to direct future focus on different aspects of the disorder including psychosocial factors and burden of disease. The study points to the fact that epidemiological data to assess the magnitude of OCD as well as other psychiatric morbidity is highly essential for future planning and development to address such problem. There is a dire need to continue further research, planning and development of programs for better understanding and management of OCD as well as other mental disorders on primary, secondary and tertiary levels. The findings of the study reflect the requirement to allocate resources at government level to explore psychiatric and psychosocial needs of the patients suffering with mental disorders. This will pave the way to improve and enhance the existing resources and create new mental health facilities for the public not only in urban but also in rural vicinities.

## CONCLUSION

Obsessive Compulsive Disorder is not a rare psychiatric and psychological phenomenon. The study highlights the need for structured and computerized record of data which can be an important and basic source for many future researches. Research highlights the need of intervention at primary and secondary level to create public awareness about OCD in particular and other mental disorders in general. Further there is also a need to improve the available mental health services and to extend them to Basic Health Units (BCO) at local level to facilitate general public.

### Authors Contribution

**SJ:** Conceived, designed, research, did data collection, statistical analysis & editing of manuscript.

**RK:** Did review and gave final approval of the manuscript.
